# Regional Prevalence of Short Stature in Saudi School-Age Children and Adolescents

**DOI:** 10.1100/2012/505709

**Published:** 2012-03-12

**Authors:** Mohammad I. El Mouzan, Abdullah S. Al Herbish, Abdullah A. Al Salloum, Ahmad A. Al Omer, Mansour M. Qurachi

**Affiliations:** ^1^Department of Pediatrics, King Saud University, P.O. Box 2925, Riyadh 11461, Saudi Arabia; ^2^The Children's Hospital, King Saud Medical Complex, Ministry of Health, P.O. Box 2897 Riyadh 11196, Saudi Arabia; ^3^Department of Pediatrics, Al Yamama Hospital, Ministry of Health, P.O. Box 60989 Riyadh 11555, Saudi Arabia

## Abstract

*Objective*. To assess the magnitude of regional difference in prevalence of short stature in Saudi children and adolescents. *Subjects and Methods*. A representative sample from three different regions of the Kingdom of Saudi Arabia (KSA) (North, Southwest, and Center) was used to calculate the prevalence of short stature (standard deviation score less than −2) in children 5 to 17 years of age. *Results*. There were 9018 children and adolescents from 5 to 17 years of age (3366, 2825, and 2827 in the Northern, Southwestern and Central regions, resp.) and 51% were boys. In both school-age children and adolescents, there was a significantly higher prevalence of short stature in the Southwestern than in the Northern or the Central region (*P* < 0.0001). *Conclusion*. The finding of significant regional variation between regions helps in planning priorities for research and preventive measures.

## 1. Introduction

Short stature which is the result of poor linear growth is a common referral of children and adolescents to specialized clinics for investigations. Short stature may be part of well-known syndromes, systemic and endocrine diseases, or an early presentation of treatable conditions such as isolated growth hormone deficiency and celiac disease and inflammatory bowel disease [1–4]. However, the more common isolated, idiopathic short stature in “normal” children is thought to be related more to environmental factors such as chronic under nutrition [5]. Knowledge of the prevalence of short stature is the first step for prevention of this condition and its complications. Although national prevalence data are important, significant regional variations in the prevalence of nutritional disorders such as malnutrition in preschool children have been reported which resulted in recommendations for priorities for further research and preventive programs for regions with high prevalence of malnutrition [6]. These findings suggest that regional variation in prevalence of other nutritional disorders such as short stature may also exist in school-age children and adolescents. Therefore, the objective of this report is to assess the regional variations in prevalence of short stature in the Kingdom of Saudi Arabia.

## 2. Methods

The prevalence of short stature in Saudi school-age children and adolescents (5–17 years of age) was calculated from the data set of the national Saudi reference. The design and methodology of the survey used to establish the latter reference have been reported in details elsewhere [7]. In brief, a multistage probability procedure was used to randomly select a cross-sectional sample from a stratified listing of the population of the KSA that was available at the time of study design. Therefore, the sample was representative of all the socioeconomic strata including weighted urban rural representation in each region. House-to-house visits were made to all selected houses where a survey questionnaire, clinical examination, and body measurements were completed by primary care physicians and nurses. Stature measurements were performed on all healthy children and adolescents by trained physicians and nurses according to recommended standards [8]. A representative sample from three different regions of the KSA (North, Southwest, and Center) was used to calculate the prevalence of short stature for age for children 5 to 17 years. The 2000 Center for Disease Control and Prevention (CDC) growth reference and related software were used for the calculation of prevalence data [9]. The prevalence of short stature was defined by the proportion of children with height for age below minus 2 standard deviations (<−2 SD). Prevalence data were calculated for three regions from the North (Hail, Jouf, and Northern Borders), two Southwestern regions (Gizan and Aseer), and two Central regions of the kingdom (Riyadh and Qassim). In this study, the combinations of regions were based on population characteristics. Accordingly, the Northern region, represented by Hail, Al Jouf, and the Northern Borders, has a majority of stable tribal population, the Southwestern region represented by Aseer and Gizan also has a majority of stable tribal population different from the North, and the Central region, represented by Riyadh and Qassim, consists of a multiethnic population. Although the population in each combination, such as Riyadh and Qassim, may be considered similar, the Northern, Southwestern, and Central regions are different from each other representing the geographic and ethnic spectrum of the country. Chi-square test was used to assess the difference in prevalence between genders and regions and a *P* value of <0.05 was considered.

## 3. Results

**Figure 1:**
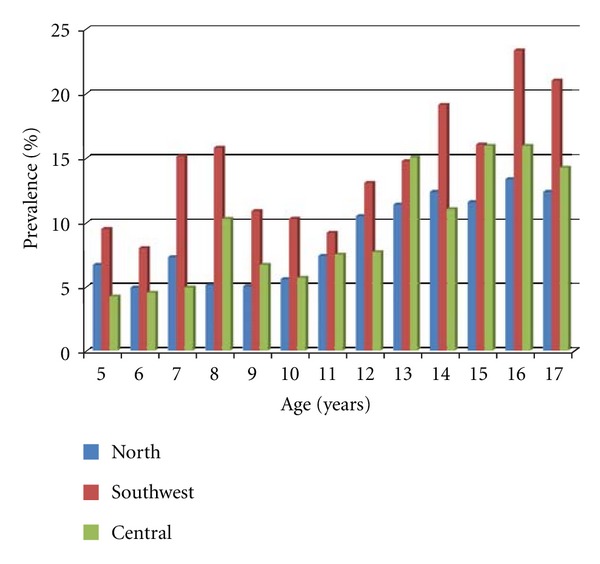
Regional variation in prevalence of short stature according to age.

The total sample size was 9018 children and adolescents from 5 to 17 years of age (51% boys). The regional distribution of the children was 3366, 2825, and 2827 in the Northern, Southwestern, and Central regions, respectively. The prevalence of moderate short stature in school-age children is presented in Table 1 indicating a significantly higher prevalence in the Southwestern than in the Northern or the Central region (*P* < 0.0001). The prevalence was not statistically different between the Northern and Central region (*P* = 0.214). There was no significant difference in prevalence of short stature between adolescent boys and girls in the Northern (*P* = 0.949), the Southwestern (*P* = 0.670), or the Central region (*P* = 0.307). The prevalence data for adolescents are depicted in Table 2 showing a significantly higher prevalence in the Southwestern than in the Northern or the Central region (*P* < 0.0001). There was no significant difference between the Northern and Central region (*P* = 0.229). There was no significant difference in prevalence of short stature between boys and girls in the Northern (*P* = 0.328), the Southwestern (*P* = 0.731), or the Central region (*P* = 0.223). In all age groups, there is generally increasing prevalence of short stature with age in both genders and in all regions. These regional variations are summarized in Figure 1.

## 4. Discussion

The identification of regional differences of nutritional disorders such as short stature in older children and adolescents is important for targeting high-prevalence areas for programs to prevent short stature and its complications such as psychologic disorders and obesity and its complications [10–14]. In this report, there was a significantly higher prevalence of short stature in the Southwestern regions compared to the Northern or Central region which is significant and contrasts with the national prevalence of 11% [15]. Such regional variations have been reported from other countries. In a report from the West Bank, Palestine, the prevalence of stunting in school children aged 13–15 years was 9.2% and 7.3% in boys and girls, respectively, in Ramallah and 9.4% to 4.2% in boys and girls, respectively, in Hebron [16].

 In addition to genetic causes, the high altitude, the predominance of rural areas exceeding 60%, and the high prevalence of malnutrition are some of the environmental factors that have been documented in the Southwestern regions and may account at least in part for the high prevalence of short stature [6, 17]. However, further research is needed for identification of the causes and subsequently development of preventive programs.

Among other environmental factors, parental education and socioeconomic status are widely recognized contributing factors to short stature. In a previous report, we have shown that the prevalence of malnutrition was highest among children with limited parental education [18]. In Brazil narrowing socioeconomic inequalities led to reduction of prevalence of short stature [19]. In addition, in a report on stunting in Indian adolescents aged 11–16 living in South India compared with a sample from the same ethnic background but living in Dubai, United Arab Emirates (UAE), a prevalence of stunting 38.8% and 36.9% in boys and girls, respectively, who are living in India, compared to 8.9% and 11.6% for South Indian students living in the UAE [20] was revealed. These reports not only indicate the role of environmental factors but more importantly that improvement in these factors led to reduction of prevalence of short stature and its complications.

In conclusion, this report demonstrates the importance of regional variations in prevalence of short stature in planning priorities for research and prevention.

## Figures and Tables

**Table 1:** Regional prevalence of short stature in school-age children.

Age (years)	North: No (% <2 SD*)			Southwest: No (% <2 SD)			Central: No (% <2 SD)		
	Boys	Girls	All	Boys	Girls	All	Boys	Girls	All
5–<6	152 (8.6)	124 (4.8)	276 (6.7)	110 (3.6)	117 (15.4)	227 (9.5)	146 (4.8)	111 (3.6)	257 (4.2)
6–<7	131 (6.1)	140 (3.6)	271 (4.9)	112 (11.6)	93 (4.3)	205 (8)	104 (5.8)	129 (3.1)	233 (4.5)
7–<8	149 (6)	141 (8.5)	290 (7.3)	111 (13.5)	113 (16.8)	224 (15.2)	124 (6.5)	120 (3.3)	244 (4.9)
8–<9	150 (3.3)	133 (6.8)	283 (5.1)	118 (16.9)	94 (14.9)	212 (15.9)	129 (15.5)	118 (5.1)	247 (10.3)
9–<10	136 (4.4)	145 (5.5)	281 (5)	124 (9.7)	108 (12)	232 (10.9)	131 (6.9)	109 (6.4)	240 (6.7)
10–<11	152 (6.6)	134 (4.5)	286 (5.6)	101 (7.9)	118 (12.7)	219 (10.3)	114 (5.3)	131 (6.1)	245 (5.7)
11–<12	145 (5.5)	130 (9.2)	275 (7.4)	113 (14.2)	98 (4.1)	211 (9.2)	112 (8.9)	116 (6)	228 (7.5)
12–<13	123 (12.2)	126 (8.7)	249 (10.5)	106 (12.3)	116 (13.8)	222 (13.1)	117 (9.4)	102 (5.9)	219 (7.7)

Overall	1138 (6.5)	1073 (6.4)	2211 (6.5)	895 (11.3)	857 (12)	1752 (11.6)	977 (6)	936 (4.9)	1913 (5.5)

*Standard deviation.

**Table 2:** Regional prevalence of short stature in adolescents.

Age (years)	North: No (% <2 SD*)			Southwest: No (% <2 SD)			Central: No (% <2 SD)		
	Boys	Girls	All	Boys	Girls	All	Boys	Girls	All
13–<14	110 (9)	116 (13.8)	226 (11.4)	117 (12.8)	126 (16.7)	243 (14.8)	106 (8.5)	97 (21.6)	203 (15.1)
14–<15	153 (10.5)	127 (14.2)	280 (12.4)	125 (20)	109 (18.3)	234 (19.2)	86 (7)	112 (15.2)	198 (11.1)
15–<16	115 (14.8)	107 (8.4)	222 (11.6)	95 (12.6)	102 (19.6)	197 (16.1)	97 (15.5)	85 (16.5)	18 (16)
16–<17	115 (10.4)	104 (16.3)	219 (13.4)	115 (21.7)	103 (25.2)	218 (23.5)	93 (20.4)	78 (11.5)	171 (16)
17–<18	98 (11.2)	110 (13.6)	208 (12.4)	88 (25)	93 (17.2)	181 (21.1)	68 (13.2)	72 (15.3)	140 (14.3)

Overall	591 (11.2)	564 (13.3)	1155 (12.2)	540 (18.3)	533 (19.3)	1073 (18.8)	450 (12.9)	444 (16.2)	894 (14.5)

*Standard deviation.
